# A Display Filter Alters SEF95 Calculation in the BIS Monitor: A Persistent Design Flaw with Clinical and Research Implications

**DOI:** 10.1097/ALN.0000000000005762

**Published:** 2025-10-28

**Authors:** Darren Hight, Heiko A. Kaiser, Peter J. Schuller

**Affiliations:** 1 University of Bern, Bern, Switzerland; 2 Hirslanden Clinic Aarau, Aarau, Switzerland; 3 Cairns Hospital, Cairns, Australia

## To the Editor:

Clinical electroencephalogram (EEG) monitors are widely used to guide anesthetic dosing, yet several contain undisclosed technical flaws that alter the recorded EEG. In some modes, the Entropy monitor (GE HealthCare, USA) exhibits uneven frequency responses and aliasing artefacts due to a basic signal processing error,^1^ and the SedLine device (Masimo, USA) alters EEG amplitude, amplitude resolution, and sample rate depending on user display settings and even clips the saved trace if it exceeds the vertical display axis.^2^ These are not minor quirks but fundamental technical flaws in devices used to guide anesthetic care and generate research data. We describe a similar long-standing error in the Bispectral Index (BIS) monitor (Medtronic, Ireland) that affects the calculation of the spectral edge frequency 95 (SEF95).

The SEF95 is a standard EEG metric, defined as the frequency below which 95% of spectral power lies,^3,4^ and is calculated over the full clinical bandwidth (0.5 to 30 Hz, per BIS documentation).^4^ It typically decreases with increasing anesthetic effect and has been used in both research and clinical monitoring for decades.^5–8^

In the BIS series of EEG devices, the user-selectable screen display filter affects not only the displayed waveform but also the calculation of SEF95. The filter removes frequencies under 2 Hz, thereby falsely elevating the SEF95, suggesting that patients are more lightly anesthetized than they are. This is in contrast to the product information manual, which states that the SEF95 is calculated (appropriately) from the 0.5- to 30-Hz frequency band. It appears that the BIS itself is not affected, although we have not performed an exhaustive study. Reverse engineering suggests that the SEF95 does not seem to play a major role in the BIS algorithm.^9^

The SEF95 should not vary with user interface settings any more than the BIS itself and especially not when such behavior is undocumented. This represents monitor processing, not patient physiology. We have confirmed that this error is present in multiple BIS generations, including the A-1050 (late 1990s), A-2000 (early 2000s), and BIS Vista (all from Aspect Medical Systems, Inc., USA), and the current BIS Advance (Medtronic). Because the filter is enabled by default in current devices, the problem is widespread.

**Fig. 1.**
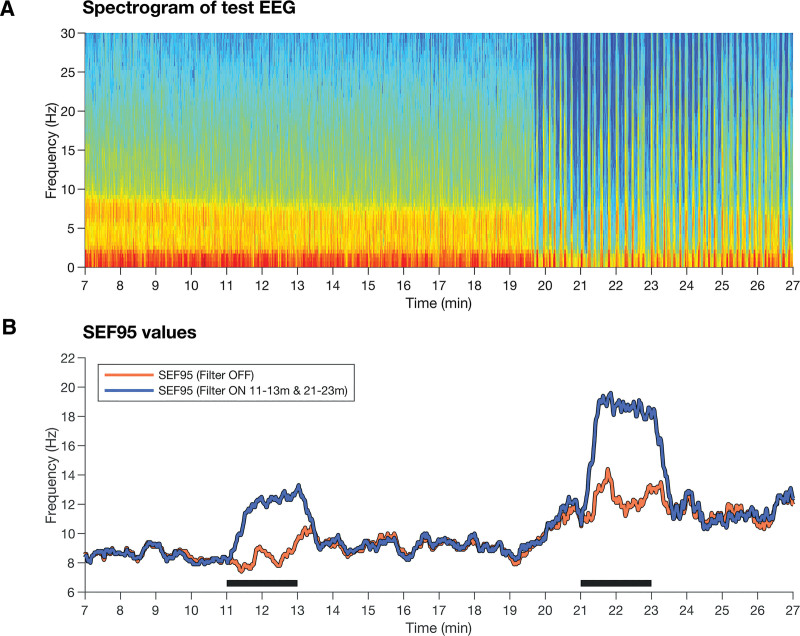
Effect of the Bispectral Index (BIS) display filter on spectral edge frequency 95 (SEF95) calculation. (*A*) Spectrogram of the test electroencephalogram (EEG). Burst suppression begins just before minute 20, visible as *vertical dark bands* corresponding to suppressed periods. Activating or deactivating the display filter does not affect the recorded EEG. (*B*) Time-aligned SEF95 traces exported from the BIS Vista monitor during two replays of the same EEG. In one replay (*orange trace*), the display filter remained off throughout. in the other (*blue trace*), the filter was on during minutes 11 to 13 and 21 to 23, indicated by *black bars*. The SEF95 increases substantially when the display filter is on.

To illustrate this filter effect, we replayed a 30-min EEG through a BIS Vista twice using a calibrated electronic system: once with the filter off and once with it briefly activated during an alpha–delta state (11 to 13 min) and burst suppression (21 to 23 min). When the filter was activated, SEF95 rose immediately and stabilized within approximately 30 s. With the filter off, mean SEF95 values were 8.5 Hz (alpha–delta) and 12.2 Hz (burst suppression). With the filter on, they increased by approximately 30% to 12.4 Hz and 18.5 Hz. Figure 1 illustrates the effect of the display filter on the SEF95. This case also demonstrates how the SEF95 can increase during burst suppression, likely because bursts contain more high-frequency components than the alpha–delta pattern. The display filter error exaggerates this effect, raising the SEF95 to values that suggest the patient may be inadequately anesthetized precisely when the burst suppression indicates deep unconsciousness.

There are two broad implications. First, clinical use: elevated SEF95 values give the misleading impression that patients are more lightly anesthetized than they are. Even if SEF95 is used only as a trend measure, this can be misleading because a change in display settings during a case will suggest a significant EEG change.

Second, research distortion: published studies have reported BIS-derived SEF95 values without noting whether the display filter was active.^5,6^ This is understandable, as neither clinicians nor investigators would expect a user interface setting to alter a core calculated parameter. These studies have been widely cited and their findings incorporated into reference texts.^10^

The problem could then be compounded. Misleading reference values, in turn, could lead to future clinicians *underdosing* patients if they are using a monitor that generates accurate values. In this context, we would encourage Medtronic to correct this issue and suggest that they notify users that SEF95 values are affected by the display filter setting.

More broadly, the persistence of such flaws contrasts starkly with pharmaceuticals. A clear error in a standard drug preparation would be detected during testing and corrected or lead to prompt withdrawal if discovered later. By contrast, this SEF95 error has persisted across BIS generations for more than two decades, suggesting to us that these devices are not subjected to the kind of systematic validation routinely required for drugs. If such devices are to influence pharmacologic dosing, it seems reasonable to expect similar standards of quality assurance and regulatory oversight as for the drugs themselves.

## Research Support

Support was provided solely from institutional and/or departmental sources.

## Competing Interests

Dr. Hight has received speaking honoraria from Medtronic (Minneapolis, Minnesota). The other authors declare no competing interests.
